# Quantitative trait loci affecting the 3D skull shape and size in mouse and prioritization of candidate genes *in-silico*

**DOI:** 10.3389/fphys.2015.00092

**Published:** 2015-03-26

**Authors:** A. Murat Maga, Nicolas Navarro, Michael L. Cunningham, Timothy C. Cox

**Affiliations:** ^1^Division of Craniofacial Medicine, Department of Pediatrics, University of WashingtonSeattle, WA, USA; ^2^Center for Developmental Biology and Regenerative Medicine, Seattle Children's Research InstituteSeattle, WA, USA; ^3^Laboratoire PALEVO, Ecole Pratique des Hautes EtudesDijon, France; ^4^UMR uB/CNRS 6282 – Biogéosciences, Université de BourgogneDijon, France; ^5^Department of Anatomy and Developmental Biology, Monash UniversityClayton, VIC, Australia

**Keywords:** skull shape, geometric morphometrics, 3D imaging, candidate gene enrichment, multivariate QTL mapping

## Abstract

We describe the first application of high-resolution 3D micro-computed tomography, together with 3D landmarks and geometric morphometrics, to map QTL responsible for variation in skull shape and size using a backcross between C57BL/6J and A/J inbred strains. Using 433 animals, 53 3D landmarks, and 882 SNPs from autosomes, we identified seven QTL responsible for the skull size (SCS.qtl) and 30 QTL responsible for the skull shape (SSH.qtl). Size, sex, and direction-of-cross were all significant factors and included in the analysis as covariates. All autosomes harbored at least one SSH.qtl, sometimes up to three. Effect sizes of SSH.qtl appeared to be small, rarely exceeding 1% of the overall shape variation. However, they account for significant amount of variation in some specific directions of the shape space. Many QTL have stronger effect on the neurocranium than expected from a random vector that will parcellate uniformly across the four cranial regions. On the contrary, most of QTL have an effect on the palate weaker than expected. Combined interval length of 30 SSH.qtl was about 315 MB and contained 2476 known protein coding genes. We used a bioinformatics approach to filter these candidate genes and identified 16 high-priority candidates that are likely to play a role in the craniofacial development and disorders. Thus, coupling the QTL mapping approach in model organisms with candidate gene enrichment approaches appears to be a feasible way to identify high-priority candidates genes related to the structure or tissue of interest.

## Introduction

Understanding the development and evolution of organismal form requires knowledge of its size and the nature of genetic variation. This genetic variation can stem from any gene whose product is involved in the developmental processes that form the structure of interest. In this context, quantitative trait loci (QTL) are genetic loci, alleles of which contribute to the variation in a quantitative trait (Broman and Sen, 2009). Generally, quantitative traits are multifactorial and are influenced by several polymorphic genes and environmental conditions, so one or many QTL can influence any given trait or phenotype. As a model of complex morphological structure the mouse mandible and its molars have been the focus of several QTL mapping studies that investigated its genetic architecture, imprinting effects, integration and modularity (Klingenberg et al., 2001, 2004; Workman et al., 2002; Leamy et al., 2008; Suto, 2009; Boell et al., 2011, 2013; Boell, 2013). Skeletal features affecting body size in mice have also been investigated (Kenney-Hunt et al., 2006, 2008; Norgard et al., 2009). In contrast, the skull has received somewhat less attention. One reason for the lack of attention is the complexity of the genotype-phenotype mapping of this structure (Hallgrimsson et al., 2014). Compared to the more simplistic model system of the mandible (Klingenberg and Navarro, 2012), many genetic and epigenetic processes interacting at different times in development are required to maintain coordinated growth of the head (Lieberman, 2011; Hallgrimsson et al., 2014). These interactions, in turn, may obscure the direct genotype-phenotype association. However, some studies have been carried out successfully. Leamy et al. identified 26 QTL involved in phenotypic variation in the skull from a population derived from the F2 generation of the intercross between LG/J and SM/J strains (Leamy et al., 1999). Kenney-Hunt et al. used the same population and measured 70 skeletal traits, 12 of which were craniofacial (CF) measurements (Kenney-Hunt et al., 2008). They identified 781 skeletal QTL by mapping every measurement individually; 105 of which were pleiotropic, meaning they affected two or more traits. Of these pleiotropic loci, 65 harbored at least one craniofacial trait. Again using the same intercross and the same set of measurements, Wolf et al. investigated the genetic architecture of covariation in the skull traits and found that the integration is achieved by a complex combination of pleiotropic effects (Wolf et al., 2005). Nishimura et al. reported the presence of two QTL that affect zygomatic arch width and three QTL for snout length using F2 mice from a C57BL/6J and DBA/2J intercross (Nishimura et al., 2003). Instead of linear measurements, Burgio et al. used 26 cranial landmarks, collected separately from dorsal and ventral surfaces, and interspecific recombinant congenic strains (IRCs) (Burgio et al., 2009). Although neither the number nor the location of QTL were described, they reported finding multiple QTL, some of which were consistent with the findings of Leamy et al. (1999). A recent genome-wide association study (GWAS) focused on the 3D skull shapes of wild-caught mice (Pallares et al., 2014). Using principal components (PC), the authors identified nine loci with high precision, most of which were within a megabase. Few loci were consistent with previously reported findings from Burgio et al. (2009). Although the authors reported that ~64% of the skull shape variance has a genetic basis, these loci together explain only 13% of the total shape variance. This variation nevertheless was spread across all autosomes, in a manner generally proportional to chromosome length, underlining the highly polygenic nature of craniofacial shape (Pallares et al., 2014).

Outside of genus *Mus*, Schoenebeck et al. used a GWAS approach and identified 5 QTL responsible for skull shape variation in domestic dogs (Schoenebeck et al., 2012). For at least one of these QTL, a likely causal variant (in BMP3) was identified. In primates, two studies also used GWAS and linear measurements from lateral cephalograms to map QTL responsible for the CF traits in baboons and humans, respectively (Sherwood et al., 2008, 2011). In a recent human GWAS, five candidate genes affecting facial shape variation in Europeans were identified (Liu et al., 2012).

Despite the insight they provide, the limitation of the majority of these studies has been the approach employed to quantify and describe the variation in their traits of interest. Most relied on linear measurements to define the shape of a complex structure like the mandible or skull. The few studies that preferred geometric morphometrics (or landmark based) approaches over traditional morphometrics either used only 2D landmarks from one side of the structure (Klingenberg et al., 2001, 2004), or partitioned the phenotype into different components such as dorsal and ventral (Burgio et al., 2009) due to an inability to capture the entire shape in 3D. Others simply mapped each PC separately due to the complexity of properly handling population structure (Pallares et al., 2014).

Over the last decade, significant advances have been made in 3D imaging and the analysis of 3D shape. Here, we describe the first application of high-resolution 3D micro-computed tomography (microCT), together with 3D landmarks and geometric morphometrics, to map QTL responsible for variation in skull shape and size using a backcross between two common inbred strains of mice. In addition, we have incorporated a higher density genotyping approach than previously attempted with an intercross of inbred strains. We also used a bioinformatics approach to identify high priority CF candidate genes within the identified QTL intervals. Finally, we present a new QTL analysis package for R statistical software that is more suitable for the mapping of shape as a multivariate feature.

## Materials and methods

### Experimental design

Three C57BL/6J males and three A/J females were used to derive F1 generation backcrossed to A/J males and females. All founder animals were acquired from the Jackson Laboratories, Maine. 163 offspring were produced from AJ (♀)×F1 (♂) backcrosses (84 females and 79 males) and 270 (128 female and 142 males) from the reciprocal F1 (♀) ×AJ (♂) crosses. All 433 animals were sacrificed at postnatal day 28, and their heads preserved at −20°C for *ex-vivo* imaging. Liver tissue was also collected from each animal for DNA extraction using a salt-chloroform extraction procedure followed by ethanol precipitation (Seto et al., 2007). All animal protocols were approved by the University of Washington's Institutional Animal Care and Use Committee.

For genotyping, isolated DNA was hybridized to a commercially available linkage panel (http://www.illumina.com/products/mouse_md_linkage.ilmn). This panel consists of 1449 SNPs selected from the Wellcome-CTC Mouse Strain SNP Genotype Set and was designed to provide uniform genome distribution at a density of approximately three SNPs per 5 Mb across the genome. Genotyping was conducted at the Northwest Genomic Center at the University of Washington. Non-polymorphic loci and the X-chromosome markers were removed, leaving 882 informative SNPs.

### 3D imaging and geometric morphometrics

**Figure 1:**
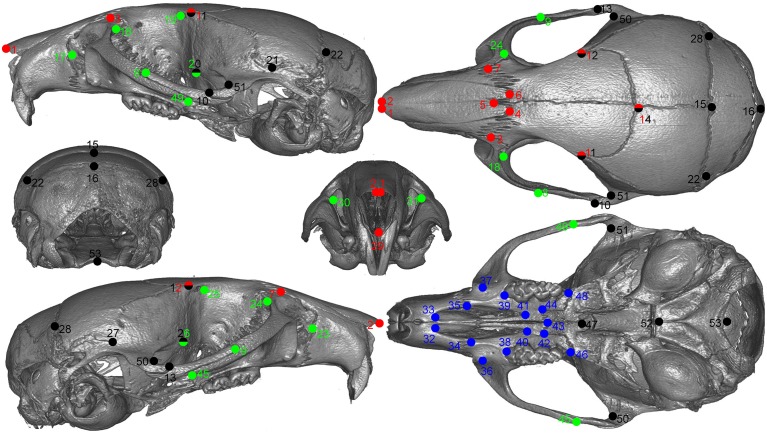
**Landmarks used in the study**.Green: lateral face, red: dorsal face, black: neurocranium, blue: palate. Points with two colors are assigned to both regions.

All animals were imaged at the Small ANimal Tomographic Analysis (SANTA) Facility at Seattle Children's Research Institute using high-resolution microcomputed tomography (model 1076; Skyscan, Belgium) employing a standardized imaging protocol (18 μm spatial resolution, 0.5 Al filter, 55 kV, 420 ms exposure, 3 frame averaging). Reconstructed image stacks were loaded into 3D Slicer (http://www.slicer.org) and rendered in 3D. A random subset of 50 samples was landmarked twice using an initial set of 55 skull landmarks. We calculated the difference in the coordinates of matching landmarks from the two sets (i.e., observer error) and removed those that consistently exceed an arbitrary cut of 7 voxels (0.125 mm). Based on these results, two landmarks were dropped from the set. The remaining samples were landmarked only once for efficiency. Figure 1 shows the final set of landmarks used in the study.

For this study, biological shape is defined as the geometry that remains after the size, location, orientation (Kendall, 1984), and as well as any departure from perfect bilateral symmetry is removed from the landmark data (Mardia et al., 2000). Asymmetry can arise from developmental perturbations due to non-genetic factors and potentially can obscure the genotype-phenotype mapping. So, handling symmetry of structures properly is an important statistical issue in all studies of structures with internal symmetry (Klingenberg et al., 2002). A full generalized Procrustes analysis (Dryden and Mardia, 2008) with object symmetry (Mardia et al., 2000; Klingenberg et al., 2002) was performed on these 3D landmarks using MorphoJ (Klingenberg, 2011). There had been a debate on the consistency of the results produced by the Procrustes based superimposition and alternative morphometric methods using landmarks, such as Euclidean Distance Matrix Analysis, were proposed (Lele and Richtsmeier, 1990, 1991; Richtsmeier et al., 2002). However, further statistical and simulation studies demonstrated that the Procrustes-based approaches outperformed alternative methods (Kent and Mardia, 1997; Rohlf, 2000a,b, 2003; Adams et al., 2013).

We use the centroid size, the square root of the sum of squared Euclidean distances from each landmark to their own centroid, as a proxy for overall skull size (Dryden and Mardia, 2008). After superimposition of both the original and mirrored copy of landmark configurations, and orthogonal projection onto the shape tangent space, the symmetric component of shape variation was extracted by averaging the original and relabeled reflected copy of the landmark configurations using the appropriate procedures in MorphoJ (Klingenberg, 2011). Shape must be understood and treated as a multivariate trait of high dimensionality, but after removing the effect of size, translation, rotation, and asymmetry some of the dimensions of the shape space remain invariant because of the constraints. In our study, the effect of any factor is a multi-dimensional vector representing the direction and magnitude of the shape change of the overall configuration of landmarks within the shape space.

### Mapping shape loci

The effect of the additive shape QTL at locus *l* was estimated using Haley-Knott regression (Haley and Knott, 1992; Knott and Haley, 2000; Haley and Knott, 1992; Knott and Haley, 2000) by fitting the multivariate linear model ***y_i_* | *M_i_* ~ *N_q_* (μ + ∑_*c*_*x_ic_* β_*c*_ + ∑_*j*_*p_ij_* β_*j*_, S)** where *x_ic_* is the value of the covariate *c* and *p*_ij_ = Pr (*g*_i_ = *j*|**M***_i_*) is the probability of the QTL genotypes given the flanking markers for individual *i* and the **β** are the *q*-dimensional effect of the covariate *c* or of the genotype *j*. These probabilities were computed using R/qtl (Broman et al., 2003) at each centimorgan along the 19 autosomes considering a genotyping error rate of 10^−4^ and a Carter-Falconer map function, which provides a low estimate to the level of interference found in mice (Broman et al., 2002). Sex-averaged genetic distances were obtained from the Jackson Laboratories Mouse Converter (http://cgd.jax.org/mousemapconverter/) using marker ID and the genetic map reported by Cox et al. (2009).

The presence of any QTL was evaluated using the Pillai trace criterion (Pillai, 1967). The probabilities associated with its approximated *F* statistics were transformed to their negative log_10_ to make results comparable with LOD scores (Leamy et al., 2008). Rank deficiency related to the Procrustes superimposition must be handled at that step using either Moore-Penrose generalized inverse or using PCs with a non-zero eigenvalue, the two approaches being equivalent. We took the latter approach because of the large difference in the number of variables to treat in computation. Geometric morphometrics provides a high dimensional dataset where many dimensions may have small variances, typically an unwanted property for ratio statistics based on determinants. Moreover, Pillai's trace criterion is said to be more powerful when effects are spread over dimensions (Olson, 1976, 1979; Tabachnick and Fidell, 2007), and more robust than other classic multivariate statistics especially when data are unbalanced (Olson, 1976, 1979; Tabachnick and Fidell, 2007).

All computations for shape QTL mapping were conducted in the R/shapeQTL package written by one of the authors (NN) and available by request. The log-transformed centroid size was analyzed in a similar manner to shape using Haley-Knott regression but using R/qtl v1.28–19 (Broman et al., 2003).

### Genome-wide significance threshold

Genome-wide significance for the presence of a QTL was evaluated using a permutation approach (Churchill and Doerge, 1994). The trait (skull shape or skull size) together with its covariables (gender, direction of the cross and the log centroid size for shape) was reshuffled among individuals whereas the original genotype probabilities were kept constant. The genome scan was repeated on these data and the maximal score (LOD for centroid size or logP for shape) was recorded for each of 10,000 iterations. We took the 95% quantile of that distribution as the genome-wide threshold.

### Multiple QTL modeling

Prior studies on shape QTL stopped their genetic modeling when at most two QTL per chromosome were included (Leamy et al., 1999; Klingenberg et al., 2001). The rationale was that genetic resolution of a one-generation cross impairs QTL discoveries and that at most only two QTL per chromosome may be identified. New SNP maps combined with a large mapping panel may however allow a deeper search despite large linkage disequilibrium (LD). High dimensionality traits have some interesting features against that smoothing of LD since the *q*-dimensional QTL effect will quickly move away as QTL genotype probabilities change. This property should add power to identify linked QTL. Therefore we adopted an approach for model searching that included or dropped QTL without any prior knowledge on the number of QTL per chromosome following the approach developed for univariate traits by Broman and Speed (2002), Manichaikul et al. (2009).

Model comparison is based on the penalized LOD score proposed in the context of additive QTL modeling (Broman and Speed, 2002) and further extended to interactive QTL. The penalization takes the form of the product of genome-wide thresholds times the model complexity (Manichaikul et al., 2009). We followed the forward/backward algorithm developed in Broman and Sen (2009) but restricted the search to additive QTL only. We used a similar penalization but on logP. Briefly, after scanning the genome for an additional additive QTL and choosing the model with the largest criterion of model comparison, maximum likelihood positions of QTL in the model were found by iteratively scanning the genome for each QTL conditional on all other QTL and covariates until no further refinement was recorded. This forward search was repeated up to a model with 50 QTL. Then, backward elimination was performed from that model to the null model. At each step, the model with the largest value for the model comparison criterion was chosen and QTL positions refined as before. Finally, the best model was chosen among all visited models based on the largest penalized score. As the appropriateness of this model search and penalization in the context of the multivariate model is still to be explored, we also ran a stepwise regression model (results not shown) where the inclusion or elimination of QTL was decided on significance of the QTL conditional on all others QTL and covariates in the model. This model search was stopped when the best candidate to include did not reach the genome-wide threshold.

### Interval estimates of QTL locations

Bootstrapping does not perform very well in the context of high linkage disequilibrium and a sparse marker map such as found in the QTL mapping of F2 intercrosses or backcrosses (Manichaikul et al., 2006). To date, most shape QTL studies (Leamy et al., 1999; Klingenberg et al., 2001) have used 1-LOD support intervals (Lander and Botstein, 1989; Dupuis and Siegmund, 1999) as approximate confidence intervals. Bayes credible interval derived from the 10^LOD(θ)^ profile has been proposed as an alternative (Dupuis and Siegmund, 1999; Sen and Churchill, 2001). Coverage of these intervals has been proven stable and consistent across a variety of situations (Manichaikul et al., 2006). Both LOD-drop and Bayes intervals are nonetheless over liberal in the context of multiple QTL models because they do not account for uncertainties in the position of other QTL (Broman and Sen, 2009). Approximate confidence intervals of QTL locations for shape were then estimated from a straightforward translation to logP profile of the 10^LOD^(θ) Bayes credible intervals using the 1/*p* profile rescaled to an area underneath the curve equal to 1. Intervals were computed from the chromosome profile of the QTL conditional on all other refined QTL positions.

### QTL effects and effect size

QTL effects were estimated conditional on all covariates (size, direction of the cross and sex). Other QTL were included in the final model using Haley-Knott regression of phenotypes on the backcross parameterization of QTL genotype probabilities using the *fitqtl* function of R/qtl (Broman et al., 2003). For shape, the effect is stacking of these univariate effects. Because we used PC scores, we back transformed these multi-dimensional vectors to the tangent space. Magnitude of shape changes are commonly expressed in unit of Procrustes distance as the norm of the vector **|| β || = (ββ^*t*^)**^0.5^ with **β** being the row vector of the QTL or covariate effect (Klingenberg et al., 2001; Workman et al., 2002). The amount of variation that each term (QTL and covariates) accounts for in the multiple QTL model, given other terms in the model, was estimated as a percentage of the total Procrustes variance (Goodall, 1991). We also reported the effect size as a percentage of the projection score variance. Those scores, computed as, *s* = **yβ**^*t*^ (**ββ**^*t*^)^−0.5^, are the shape variable associated with the shape changes defined by the **β** vector and include both the effect and the residuals in that direction (Drake and Klingenberg, 2008). This percentage represents the amount of variation accounted for by any given QTL in its specific direction.

### Visualizing shape QTL effects

The effect of each shape QTL was visualized on a 3D skull model using the R/Morpho package (Schlager, 2014). For each shape QTL, the 3D surface mesh of the mean shape is deformed along the vector that defines the effect of the QTL by incremental scaling of its magnitude. The resultant animation for each shape QTL, as well as the covariates, is rendered for six anatomical views and provided as Supplementary Material.

## Results

### Skull size

Both sex and direction of the cross, but not their interaction, were found to have statistically significant effects on the skull size. The direction of the cross corresponds to whether the N2 individual is born to an F1 female or an A/J female. Males in general and N2 individuals derived from F1 female × A/J male cross have larger skulls. Although the magnitude of the difference is very small (less than 1% in both cases), because of their statistical significance both sex and direction of cross were included as covariates for the QTL mapping.

### QTL mapping for skull size

**Figure 2:**
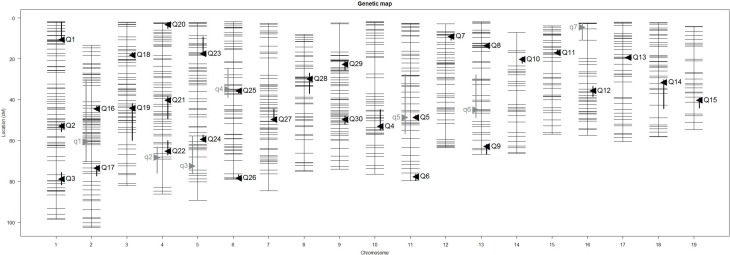
**Positions of skull size QTL (gray) and skull shape QTL (black) on genetic map**. Skull centroid size QTL (SCS.qtl) are abbreviated as “q” and are left to the chromosome map. Skull shape QTL (SSH.qtl) are as abbreviated as “Q” and are to the right of the chromosome map. Vertical bars indicate the Bayesian estimate of the confidence interval of QTL.

Our interval mapping has identified seven QTL responsible for the variation in skull centroid size (SCS.qtl). Location, nearest marker, and the confidence intervals for the identified loci, and the QTL effect are provided in Table 1. The size of the confidence intervals for these loci is highly variable; when converted to genomic location, they vary from 13 to 89 MB. The location of the SCS.qtl peaks are shown on Figure 2. SCS.qtl 0.7 on chromosome 13 shows the largest effect for increased skull size, whereas SCS.qtl 0.3 on chromosome 5 is the only QTL related to reduced skull size.

**Table 1:** **Skull centroid size QTL (SCS.qtl) identified in this study together with their logarithm of odds (LOD), Bayesian estimates of their confidence intervals (Lower CI, Upper CI), and their estimated QTL effect**.

**Size QTL**	**Closest Marker**	**Chr**	**Pos (cM)**	**LOD**	**Lower CI (bp)**	**Upper CI (bp)**	**QTL Effect**
SCS.qtl1	rs3658927	2	60.37	3.33	52,795,574	141,957,105	Increase in size
SCS.qtl2	rs13478002	4	68.08	4.85	129,338,356	142,748,609	Increase in size
SCS.qtl3	rs13478540	5	72.26	4.71	117,927,219	137,110,565	Decrease in size
SCS.qtl4	rs13478841	6	34.74	5.42	51,455,318	87,816,657	Increase in size
SCS.qtl5	rs13481127	11	48.5	4.41	45,970,896	91,694,862	Increase in size
SCS.qtl6	mCV24625340	13	44.75	7.81	53,171,098	94,326,861	Increase in size
SCS.qtl7	rs4160288	16	4.43	4.6	4,326,565	16,886,506	Increase in size

*POS column indicates the position of the peak of the estimated QTL in genetic distances. Genomic locations of the confidence intervals are based on the mouse reference genome (GRC38). Closest Marker provides the ID of the known marker closest to the peak*.

### Skull shape

**Figure 3:**
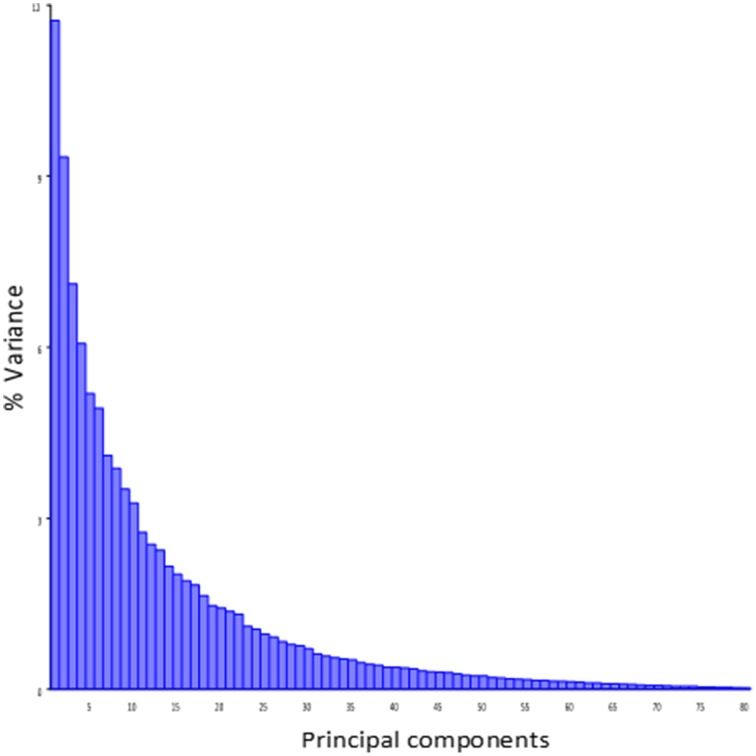
**Variance explained by the individual PCs of the symmetic component of full tangent coordinates**.

**Figure 4:**
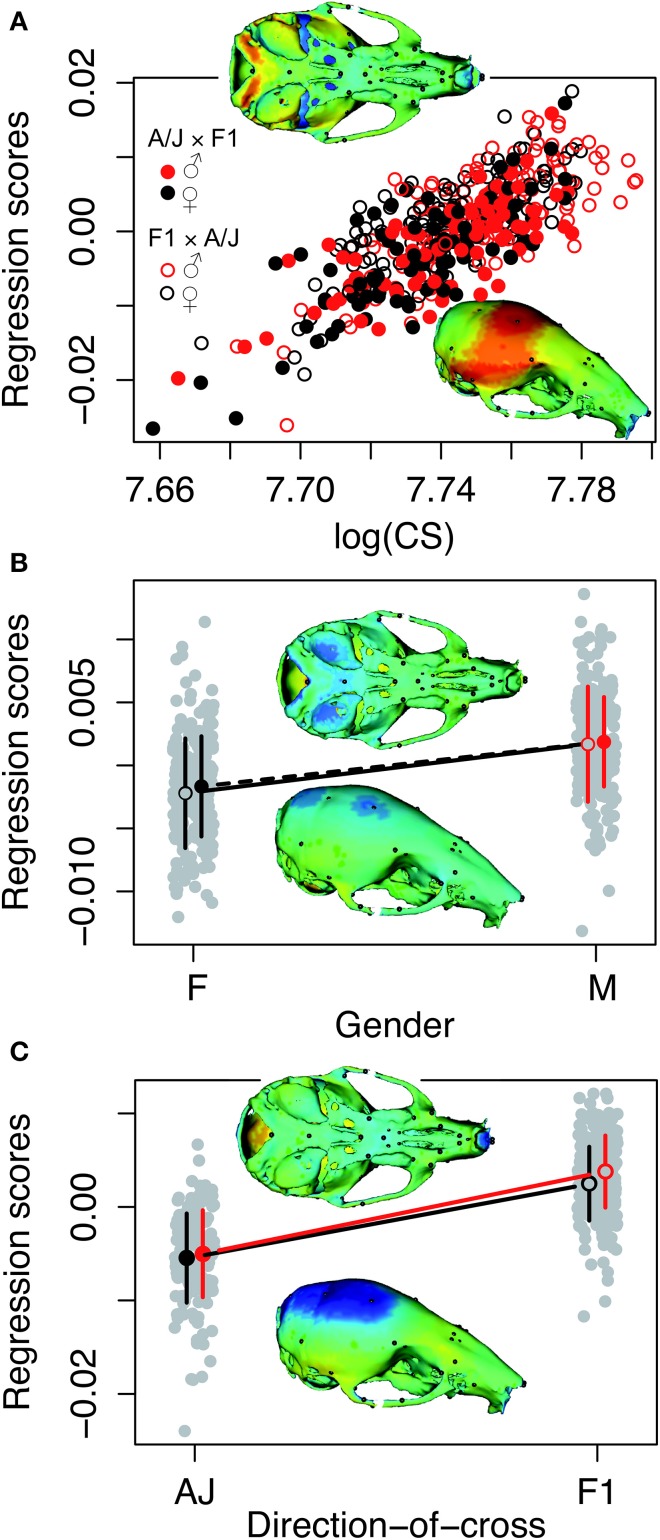
**Visualizations of the main effects of skull size, gender and directionality of the cross on skull shape**. The model is the shape resulting from the addition of the effect to the mean shape. Color map of 3D model corresponds to the deformation distance between this shape and the mean shape. Warm colors indicate shrinkage, cold colors indicate expansion with respect to the mean shape. Heatmaps are NOT to the same scale, as each effect scaled different. Dynamic visualizations of these effects can be found in the Supplemental Animations 1–3. **(A)** Skull centroid size: Increases in skull size expands the anterior brain case both vertically and laterally. **(B)** Sex: Sexual dimorphism is low and appears to map mainly on the basicranium. **(C)** Direction-of-cross: This effect shows offspring born to F1 hybrid mothers as having a higher neurocranium than those produced from pure A/J dams.

Principal component analysis of the symmetric component of the full tangent coordinates resulted in 80 PCs with non-zero eigenvalues. The amount of variation explained by each PC is shown on Figure 3. No single PC explained more than 12% of the phenotypic variation. The main effects of skull size, gender and directionality of the cross on skull shape were found to be significant (*p* < 0.0001) and explained about 1–4% of the total Procrustes variance. No significant interactions among them were found, therefore only the main effects were included in following genetic analyses as additive covariates. Increases in skull size expand the anterior brain case both vertically and laterally (Figure 4A). Sexual dimorphism is low and appears to map mainly on the basicranium (Figure 4B). The direction-of-cross effect shows offspring from F1 dams as having a higher neurocranium than those produced from A/J dams (Figure 4C). Dynamic visualizations of effects of these covariates on skull shape are provided with online Supplemental Data. To reduce the computation time, we opted to use 80 non-zero PCs in our QTL mapping instead of using 159 tangent coordinates. Since the PCA is simply a rotation along orthogonal axes, no variation is lost and PC scores can be back converted to tangent coordinates without loss of variation.

### QTL mapping for skull shape

**Figure 5:**
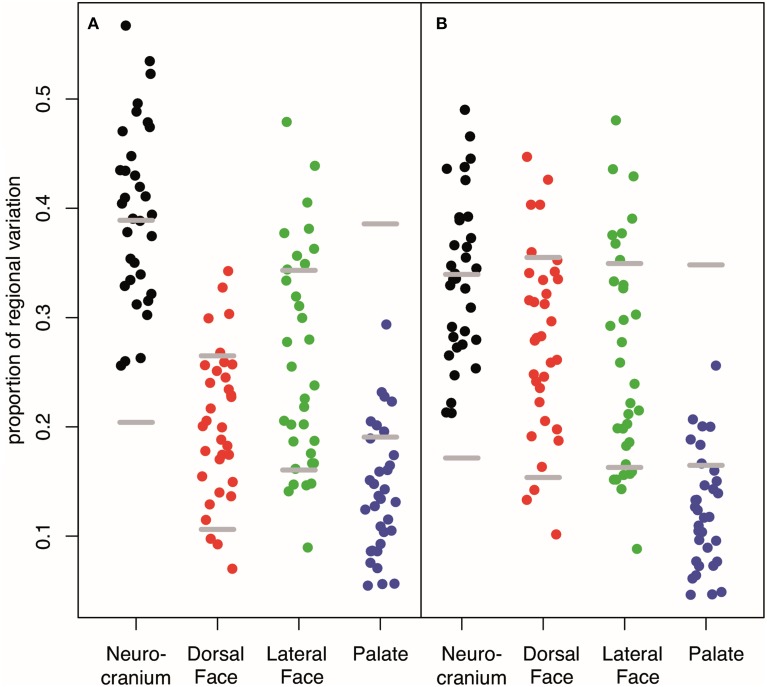
**Contribution of each anatomical region to the variation in covariates and SSH.qtl. (A)** Unstandardized **(B)** weighted by the number of landmarks in a given region. Low proportion of Dorsal Face appears to be an artifact to its low number of landmarks whereas the low proportion accounted by the palate is robust. Horizontal gray lines represent 95% intervals obtained from random vectors. Many QTL have a stronger effect on the neurocranium than expected from a random vector that will parcellate uniformly across the four cranial regions. On the contrary, most of QTL have an effect on the palate weaker than expected. For covariate or QTL specific breakdown of regional variation, see Supplemental Figure 4.

Our interval mapping has identified 30 QTL responsible for variation in skull shape (SSH.qtl). All autosomes harbor at least one, and in some cases up to three SSH.qtl (Chr 1 and 4). Location, nearest marker, and the confidence intervals for the identified SSH.qtl are provided in Table 2 and plotted on Figure 2. On average, the width of the confidence intervals was 5.4 cM (or 10.4 MB). Visualizations of each SSH.qtl are rendered in six anatomical views and are provided with online Supplemental Data. To obtain a broad sense of importance of anatomical regions involved in each SSH.qtl effect, we assigned each landmark to one of the four anatomical regions (neurocranium, dorsal face, lateral face, and palate), summed up the magnitude of displacement for each landmark in the region and visualized it as a proportion of the total magnitude of displacement. If a landmark sits on a boundary of these regions (e.g., landmark on the triple junction of sutures between the frontal, parietal and squamosal bones), the magnitude of the displacement is split equally between the bounding region (in this case lateral face and neurocranium). We also calculated a second set of ratios in which the proportions are normalized by the number of landmarks in a region. Figure 5, and also Supplemental Figure 4, show the contribution of each of these regions to a given SSH.qtl effect, as well as the additive covariates included in the analysis (size, directionality of the cross, and sex), on skull shape, with and without normalization. Even though both the neurocranium and palate have a similar number of landmarks assigned (Figure 1), it appears that the contributions of the neurocranium to the overall skull shape differences are consistently larger than those of the palate. These two regions present contributions that mostly differ (higher and lower respectively) from what can be expected from the random partitioning of landmarks (Figure 5). On the other hand, for both facial regions contributions are not different from what is expected under the null hypothesis of pleiotropy, with only a few loci having stronger than expected contributions.

**Table 2:** **Skull shape QTL (SSH.qtl) identified in this study**.

**Shape QTL**	**Closest marker**	**Chr**	**Pos (cM)**	**LOD**	**Lower CI (bp)**	**Upper CI (bp)**	**Candidate CF genes**
SSH.qtl1	mCV24784983	1	10.5	12	5,920,984	25,974,921	[Col9a1; Eya1]
SSH.qtl2	rs3678634	1	52.6	28.3	115,819,089	127,021,793	**Fcgr2b** [Gli2]
SSH.qtl3	rs13466711	1	78.6	17.7	168,066,210	175,710,316	
SSH.qtl4	rs13480734	10	52.9	18.3	89,335,908	105,325,377	[Alx1]
SSH.qtl5	rs13481127	11	48.5	27.3	80,139,712	81,911,051	
SSH.qtl6	rs3672597	11	77.5	24.8	112,611,769	114,027,932	[Sox9]
SSH.qtl7	rs3717860	12	8.8	32.5	16,866,101	28,237,591	**Taf1b**
SSH.qtl8	rs3710348	13	13.4	19.5	30,941,216	35,713,142	**Itga2**
SSH.qtl9	gnf13.115.241	13	62.8	13.4	110,281,304	118,066,332	
SSH.qtl10	rs6396829	14	20.1	28.1	30,970,692	38,186,621	
SSH.qtl11	CEL-15_43206205	15	16.8	15	39,391,647	47,392,759	
SSH.qtl12	rs4191367	16	35.4	13.9	54,657,678	66,961,706	**Epha3** [Arhgap31]
SSH.qtl13	rs6298471	17	19.2	23.8	36,252,692	43,916,321	
SSH.qtl14	rs6328845	18	31.3	11.4	56,230,871	70,306,352	**PPargc1b** [Slc26a2; Tcof1]
SSH.qtl15	rs3023496	19	40.1	17	46,867,039	49,396,956	
SSH.qtl16	rs13476580	2	44.3	20.6	72,637,926	76,844,869	
SSH.qtl17	rs6209325	2	73.3	16.5	146,817,118	155,755,548	**Pax1; Cd93** [Asxl1]
SSH.qtl18	rs6246699	3	18	14.6	34,942,416	39,449,337	
SSH.qtl19	rs4138887	3	44	13.4	96,754,992	130,100,724	[Alx3;Col11a1;Gnai3]
SSH.qtl20	rs3660863	4	3.1	9.7	3,867,296	11,352,972	[Plag1; Chd7; Gdf6]
SSH.qtl21	rs3711477	4	40.1	20.5	81,877,569	105,794,559	[Frem1]
SSH.qtl22	rs3663950	4	65.1	23.2	126,198,546	134,724,460	**Thrap3; Col16a1** [Arid1a]
SSH.qtl23	rs13459085	5	17.3	24.8	20,284,721	35,523,469	**Cad; Whsc1** [Fgfr3; Shh; Hmx1; Sh3bp2]
SSH.qtl24	CEL-5_117374791	5	59.3	14.7	117,729,579	119,888,175	**Tbx3**
SSH.qtl25	rs6181382	6	35.5	34.8	78,232,602	84,781,463	**Dok1; Gcfc2**
SSH.qtl26	rs6265387	6	78.2	27.3	144,446,852	148,260,112	**Arntl2**
SSH.qtl27	rs13479395	7	49.5	16.4	78,470,627	90,617,547	**Kif7**
SSH.qtl28	rs13479776	8	29.7	23.2	47,753,313	78,304,228	
SSH.qtl29	rs3714664	9	22.5	11.3	39,523,343	46,544,750	[Pvrl1]
SSH.qtl30	rs13480351	9	49.5	16	90,683,187	99,809,852	[Foxl2]

*Fields are same as Table 1. Genes from our list of known craniofacial genes are given in square brackets. High priority candidates genes identified in this study for their potential involvement in craniofacial development are reported in boldface*.

Effect sizes of QTL are in the order of 1% of the total Procrustes variance and show a skewed distribution with a few larger effects (Supplemental Table 1 and Supplemental Figure 2). The overall contribution of each chromosome (looking at the combined effect of QTL on the same chromosome) is in the order of 2% of the total Procrustes variance, and all together they explain up to 23% of the variance and 32% jointly with covariates, leaving about 70% of the variation unexplained. It is worth noting that the shape variation in the mapping population is spread over a large number of dimensions without strong leading directions: most PCs account for a low amount of variation and even PC1 account only about 12% of that variation (Figure 3). Similarly, specific directions of the shape space described by the QTL shape changes accounted for ~5% of the total Procrustes variance on average. However, for those specific directions, QTL account for between 5 and 30% of this variation (Supplemental Table 1). Angles between these genetic directions and phenotypic structuring (PCs) show that they are more similar to each other than two vectors drawn randomly (*p* < 0.0001), with an angle ranging from 50.7° to 69.4° (Supplemental Figure 3). This angle negatively correlates (*r* = −0.42, *p* = 0.02; Supplemental Figure 3) with the magnitude of the QTL vectors.

### Candidate craniofacial gene enrichment

We used the Jackson Laboratories Mouse Map Converter to convert our confidence interval estimates of SSH.qtl from map distances into genomic locations on the mouse reference genome (build GRCm38). We then queried the Ensembl database to obtain the list of protein coding Refseq genes for a given QTL interval. To identify the most relevant candidate genes for involvement in cranioskeletal development and phenotypic variation, we used the gene enrichment tool Toppgene from the Cincinnati Children's Hospital Medical Center. Toppgene prioritizes or ranks candidate genes based on functional similarity to a training gene list. Functional annotation-based disease candidate gene prioritization uses a fuzzy-logic based similarity measure to compute the similarity between any two genes based on semantic annotations (Chen et al., 2009). The similarity scores from individual features are combined into an overall score using statistical meta-analysis. A *p*-value for each annotation of a test gene is derived from random sampling of the whole genome (Chen et al., 2009). The utility of the Toppgene Suite was demonstrated using 20 reported GWAS-based gene–disease associations (including novel disease genes) representing five diseases, in which Toppgene ranked 19 of 20 (95%) candidate genes within the top 20% (Chen et al., 2009).

**Figure 6:**
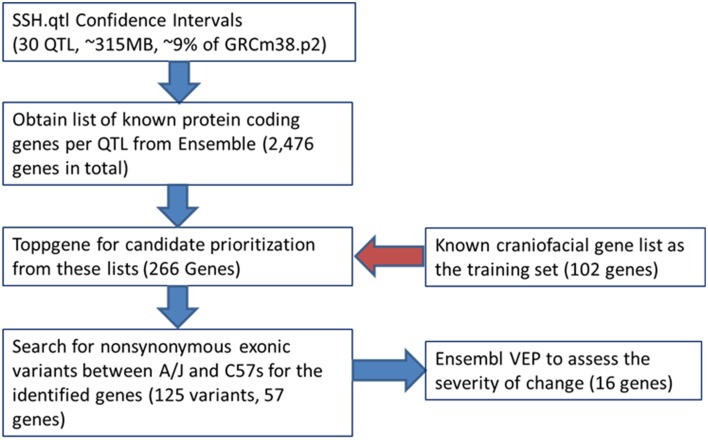
**Workflow for the bioinformatics pipeline**.

Candidate gene lists from each QTL interval were separately submitted to the gene enrichment toolkit, along with our training list of known craniofacial genes. The total training list consisted of 102 autosomal genes (Supplemental Table 2) that are known to be involved with craniofacial development and/or craniofacial disorders and was compiled by two of us (MLC and TCC). Because Toppgene uses a resampling approach (permutation test) to assess the significance of each gene, there can be potential issues due to randomness of the sampling of the genome. Therefore, candidate lists from each SSH.qtl interval were submitted to the Toppgene tool ten times. For a gene to be considered a strong craniofacial candidate, it needed to be present in all ten iterations with a significance value of 0.01 or lower. From this list, only the genes that are known to harbor exonic non-synonymous single nucleotide variants between A/J and C57BL/6J strains were retained. This information was obtained from the Wellcome Trust Mouse Genome SNP data. The list with exonic variants was submitted to the Ensembl Variant Effect Predictor (VEP) to measure the effect of variants. The resultant list contained 16 candidates with high SIFT scores (Table 2). The workflow as well as the number of candidate genes remaining at each step is provided in Figure 6.

## Discussion

Our combination of 3D geometric morphometrics, higher density genotyping (884 polymorphic SNPs), together with our multiple QTL mapping technique, enabled us to identify more than two QTL on a single chromosome with a two generation cross, specifically on chromosomes 1 and 4. This is an improvement over previous QTL mappings for skull and mandible shape and size.

### Comparison of the skull size QTL findings

The previous most comprehensive study of QTL affecting body size in mice was done by Kenney-Hunt et al. (2006), in which the size was measured through organ and necropsy weights and long bone lengths, but they did not include any cranial measurements. Of the 35 pleiotropic QTL they identified, five of them map to regions overlapping the SCS.qtls identified in this study. Three of these loci (BOD2.1, BOD4.1, BOD16.1) affect both the bone length and organ size, and two of them (ORG6.1, ORG11.1) affect only the organ size. None of our SCS.qtl map to their loci solely responsible for the long bone length, suggesting different genetic modules control the size of the skull and the long bones, unless the locus has an impact on overall body size. However, it should be noted that our population is significantly younger (28 days) than their population (70–144 days) which somewhat limits the comparisons.

In follow up work, using strictly cranial, mandibular and post-cranial measurements, and no organ or necropsy weight, Kenney-Hunt et al. identified up to 7 QTL for different skeletal traits on a single chromosome (e.g., Chr 1) (Kenney-Hunt et al., 2008). In some cases, however, the confidence intervals of these QTL overlap (e.g., all reported QTL on chromosome 1 have overlapping confidence intervals), making it difficult to argue for their independence. In other cases, their multiple QTL map to one of our SCS.qtl (e.g., skl2.03 and skl2.04 to our SCS.qtl1). Given the number of skeletal traits they found to be associated with these loci (30 and 10), this is not surprising. These loci are mostly likely involved in overall growth and are representative of a global size effect.

### Comparison of the skull shape QTL findings

There are few publications that have attempted to identify QTL responsible for variation in skull shape of mice (Leamy et al., 1999; Wolf et al., 2005; Pallares et al., 2014). The first two studies used the same F2 progeny (535 individuals) from an intercross of large (LG/J) and small (SM/J) inbred mice strains, and the same set of 76 microsatellite markers. Wolf et al was focused on epistatic interaction of the QTL responsible for the late and early developing traits, and is not directly comparable to our results (Wolf et al., 2005). In the former study, the authors were able to identify 26 QTL responsible for the measured skull characteristics (Leamy et al., 1999). Of the 26 skull QTL, 17 mapped over SSH.qtls identified in this study. However, this number could be exaggerated due to the extremely large confidence intervals of that study (27.4 cM vs. 5.4 cM in this study). At least in one case of theirs, the confidence interval spans almost the entire chromosome (QTL-13.2). The authors assigned the effect of the identified QTL on the skull characteristics either to the facial region (F) or the cranial vault (V). Of the 17 matching QTL, seven of those have a phenotypic interpretation that is consistent with the results from our study (QTL-S1.1, QTL-S1,2; QTL-S3.1; QTL-S4.1; QTL-S7.1; QTL-S8.1; QTL-S9.2). More recent study by Pallares et al. study used 178 males derived from 68 mating pairs of wild mice and very dense genotyping (Pallares et al., 2014). However, the study appears to have suffered from weak power as they identified only nine loci spread over seven chromosomes, even though they report up to 64% of the variation was heritable. This is likely due to the small number of animals used, especially with regard to the decay of linkage disequilibrium in wild populations, which implies strong association only in 100 kb blocks apart from the causal loci (Laurie et al., 2007). The failure to account for the multivariate nature of phenotypes was also a likely confounding factor as we show that directions of genetic effects may not coincide to phenotypic covariance structure (PCs) especially with natural populations where environmental variation may be large. Therefore looking only at univariate PC in such case is an approach that might have low power.

### Magnitude of effects and their mapping on the anatomical subregions of the skull

In contrast to all previous studies where adult mice of about 70 days of age or older were used, we mapped QTL for skull shape using mice aged of 28 days, an age that corresponds to approximately a week after weaning. This has important implications because masticatory muscles and mechanical load are just starting to be players in the complex development of the skull (Vecchione et al., 2010), through their effects on bone remodeling (Herring, 1993). Such epigenetic factors will channel environmental stimuli experienced by animals throughout their late ontogeny and adult life (Klingenberg and Navarro, 2012). The discovered QTL account for 23% of the total Procrustes variance in cranioskeletal shape observed between the A/J and C57BL/6J inbred strains. The direction-of-cross explains 2% of the total shape variance and partly structures PC1 (11% of its variance). Such parent-of-origin effects may arise from phenomenon such as maternal genetic effects or genomic imprinting (Hager et al., 2008; Wolf and Wade, 2009), and underlines the potential importance of maternal and other epigenetic influences on the variation of cranioskeletal form (Lieberman, 2011).

Effect sizes of QTL of skull shape variation of inbred mice appear to be small; in our study they rarely exceed 1% of the overall shape variation (Supplemental Table 1). However, they may account for up to 30% of the variation in some specific directions of the shape space. These directions correspond to specific pattern of covariation of some skull elements. For example, such patterns may correspond to the enlargement of the neurocranium (SSH.qtl7, Supplemental Animation 10), to the flattening of the cranial angle (SSH.qtl16, Supplemental Animation 19) or to the facial elongation (SSH.qtl6, Supplemental Animation 9). Their large genetic component implies the existence of important genetic constraints in the structure of the growing skull.

The compartmentalization of the QTL effects shows that in most cases contributions from neurocranium and palate on the shape variation appear different from the expectation of vectors drawn in random, but in opposite ways: A more than expected proportion of changes mapped to neurocranium, whereas the palate showed a lower than expected magnitude of changes (Figure 5). Indeed, 50% of the QTL effects show an overall effect on the skull (i.e., QTL pleiotropy across both facial regions and the neurocranium, and, even for 10%, also over the palate). Pleiotropy is the null hypothesis with geometric morphometrics data, firstly because of the mathematics behind the Procrustes superimposition (Klingenberg, 2010), but also because the highly integrated development of the skull in which even subtle changes tend to produce global effects on shape (Hallgrimsson et al., 2014).

### QTL mapping as a tool for candidate gene enrichment

In regard to our secondary goal of prioritizing the candidate genes located within QTL intervals, our approach would seem to be a feasible way to identify novel genes involved in CF development. Our SSH.qtl intervals contained 23 of the 102 already known CF genes (Table 2). Compared to the total number of candidate genes (2476) from the SSH.qtl intervals and the number of known genes on autosomes (22,644, also excludes mitochondrial genes and haplotypes) this result is highly significant (*p* < 0.0001) based on a hypergeometric test, which suggests positive enrichment of CF genes in the identified SSH.qtl intervals.

Among the 16 high priority candidates identified, some of them have recently been implicated in some craniofacial disorders, or have known expression patterns in the developing mouse craniofacial region, further supporting the validity of our approach. For example, *PAX1* is reportedly expressed in the occipital bone during fetal development (E13.5) in mice (Sonnesen et al., 2008), and also in the head mesenchyme and cranial base at E10.5 and E11.5 (Dietrich et al., 1993). Also, a mutation in *PAX1* has been proposed to cause otofaciocervical syndrome in humans (Pohl et al., 2013). *WHSC1* is a candidate gene for Wolf-Hirschhorn Syndrome, which has a distinct craniofacial phenotype including microcephaly, micrognathia, ocular hypertelorism, dysplastic ears and periauricular tags (Battaglia et al., 1993). *EPHA3* is expressed in the palatal shelves, face and middle ear of E14.5 mouse embryos (Visel et al., 2004). More recently, it has been suggested that mutations in *EPHA* family genes may cause cleft lip and palate (Agrawal et al., 2014). Mutations in the *KIF7* gene have been reported to cause Acrocallosal syndrome, which presents with a wide range of craniofacial abnormalities including macrocephaly, hypertelorism, short nose, broad nasal bridge, short philtrum with upturned upper lip, high and narrow palate (Walsh et al., 2013). *CAD* is expressed in the maxillary component of the 1st branchial arch of E10.5 mouse embryos (Rainger et al., 2012), and also weakly expressed in the nose, inner ear and the palatal shelves of E14.5 mouse embryos (Visel et al., 2004). In mice, many of the collagen genes, including *COL16A1*, are upregulated during the development of the embryonic maxillary, frontonasal and mandibular processes especially from E10.5 to E12.5 (Feng et al., 2009). In human populations, a recent GWAS study identified five genes that are responsible for facial variation observed in Europeans (Liu et al., 2012). Of these genes *COL17A1* maps to the region syntenic to our SSH.qtl15. However, *COL17A1* was not picked up as a strong candidate in our analysis due to its low SIFT score.

There are certain limitations of our candidate gene prioritization approach that need to be recognized. Arguably, the most significant limitation is the level of detail available in existing gene ontologies and other databases (such as gene expression, human/mouse phenotypes, etc) that are used in the calculation of candidate gene prioritization scores. Most genes are likely to be pleiotropic, and only a subset of their functions might be documented in these databases. This would introduce a bias and restrict what can be learned from the kind of mapping study reported here. Another related issue is the selection of “known CF genes.” Our list consists of genes that are known to cause malformations when mutated and therefore it can be considered biased toward “disease genes.” While we have not intentionally excluded non-disease gene, our rationale was that variants with lesser functional impact but in these same genes could at least conceivably contribute to natural variation in craniofacial shape. An example in support of this is *ALX1*. *ALX1* is one of our listed “CF” genes because it is known to cause clefting in humans (Uz et al., 2010). Natural variants in *ALX1* were recently reported to be responsible for almost all the phenotypic variation in beak shapes of the Galapagos finches (Lamichhaney et al., 2015), so we feel it is reasonable that expect that variants in such genes would also contribute to facial variation in mammals. While this may not be the case for all of our “known” genes, we are hopeful that with increasing information on functions and interactions of genes, this would be a self-correcting process. It is also possible that some natural variation in skull phenotype may be due to dosage effects. Indeed there are known to be strain-specific intrachromosomal rearrangements, as well as segmental deletion and duplication differences between inbred mouse strains, although in this study we did not attempt to identify all copy number variations (CNV) in our QTL regions.

Previous studies using QTL mapping of mandible and molar shape and size in mouse relied typically on 2D landmarks and sparse sampling of the genome using microsatellite markers. Our primary purpose in this study was to identify QTL responsible for the variation in skull shape and size observed in mice using 3D phenotyping and with denser genotyping, and secondarily use the identified SSH.qtl to search for important new genes involved in craniofacial development and variation. Geometric morphometrics based phenotyping appears suitable for QTL analyses because of its ability to get information from both the magnitude and the direction of the shape changes. In addition, when the focus is on the overall shape of the structure, it also has the potential to detect QTL with more precision than traditional morphometrics. However, the multivariate nature of the resultant phenotype requires specific and careful handling of its high dimensionality because its genetic sources may be largely diffused over multiple dimensions. It is also important to note that the generalized Procrustes analysis uses least-squares based optimization that assumes equal variance at all landmarks and will distribute variances across many landmarks. This is particularly troublesome if there is large variance clustered in only one or two landmarks (the Pinocchio effect), which is not the case in our dataset but still an important point to consider for every dataset. An alternative approach that does not suffer from the uncertainty introduced in the superimposition would be to use pairwise distances between landmarks as proposed by the Euclidean Distance Matrix Analysis, or EDMA, approach (Lele and Richtsmeier, 1990, 1991; Richtsmeier et al., 2002). While in theory this is an attractive approach, the major practical roadblock for this approach is the lack of availability of a sufficiently comprehensive EDMA analysis package to conduct all the morphometric analyses necessary for the QTL mapping, including the removal of asymmetry as a confounding parameter. However, there are other computational and statistical issues to consider as well. For example, the total number of interlandmark distances in our dataset with 53 landmarks is 1376. Most of those interlandmarks distances will be tightly correlated and will not be independent phenotypes in contrast to our PC scores. This will complicate the multivariate QTL mapping and calculation of the QTL effects.

In summary, we show that at least several dozen genetic variants with small effects account for a quarter of the variation observed in the sub-adult skull shape between two commonly used inbred mouse strains: C57Bl/6J and A/J. Nevertheless, some specific shape features are under strong genetic control (up to 30%). Evidence of QTL specific for some cranial regions is also shown.

The detailed follow-up bioinformatic analysis identified 16 high priority candidate genes in craniofacial development, some of them being recently implicated in craniofacial disorders. Thus, coupling the QTL mapping approach in model organisms with candidate gene enrichment approaches appears to be a feasible way to identify high-priority candidates genes related to the structure or tissue of interest.

### Conflict of interest statement

The authors declare that the research was conducted in the absence of any commercial or financial relationships that could be construed as a potential conflict of interest.
