# Supplementary material for: Quantitative trait loci affecting the 3D skull shape and size in mouse and prioritization of candidate genes in-silico
Source: Front Physiol. 2015 Mar 26;6:92. doi: 10.3389/fphys.2015.00092 (PMC4374467; doi:10.3389/fphys.2015.00092)
Supplement: Supplementary file 1 [file Presentation1.PDF]

SUPPLEMENTAL FIGURES

Supplemental Figure 1. Shape variables the most associated with QTL. **Top** is an example of QTL (SSH.qtl.6) affecting preferentially the face. **Middle** is an example of QTL (SSH.qtl.7) affecting preferentially the neurocranium. **Bottom** is an example of pleiotropic QTL (SSH.qtl.25) affecting all four components of the skull (neurocranium, dorsal and lateral faces and palate). Effects correspond to the contribution of the addition of one C57BL/6J (B6) allele. Color map of 3D model corresponds to the deformation distance between this shape and the mean shape meaning blue is bigger and red is smaller than the mean. Dynamic animations of these SSH.qtl can be found in Supplemental Animations 9, 10 and 25 respectively.

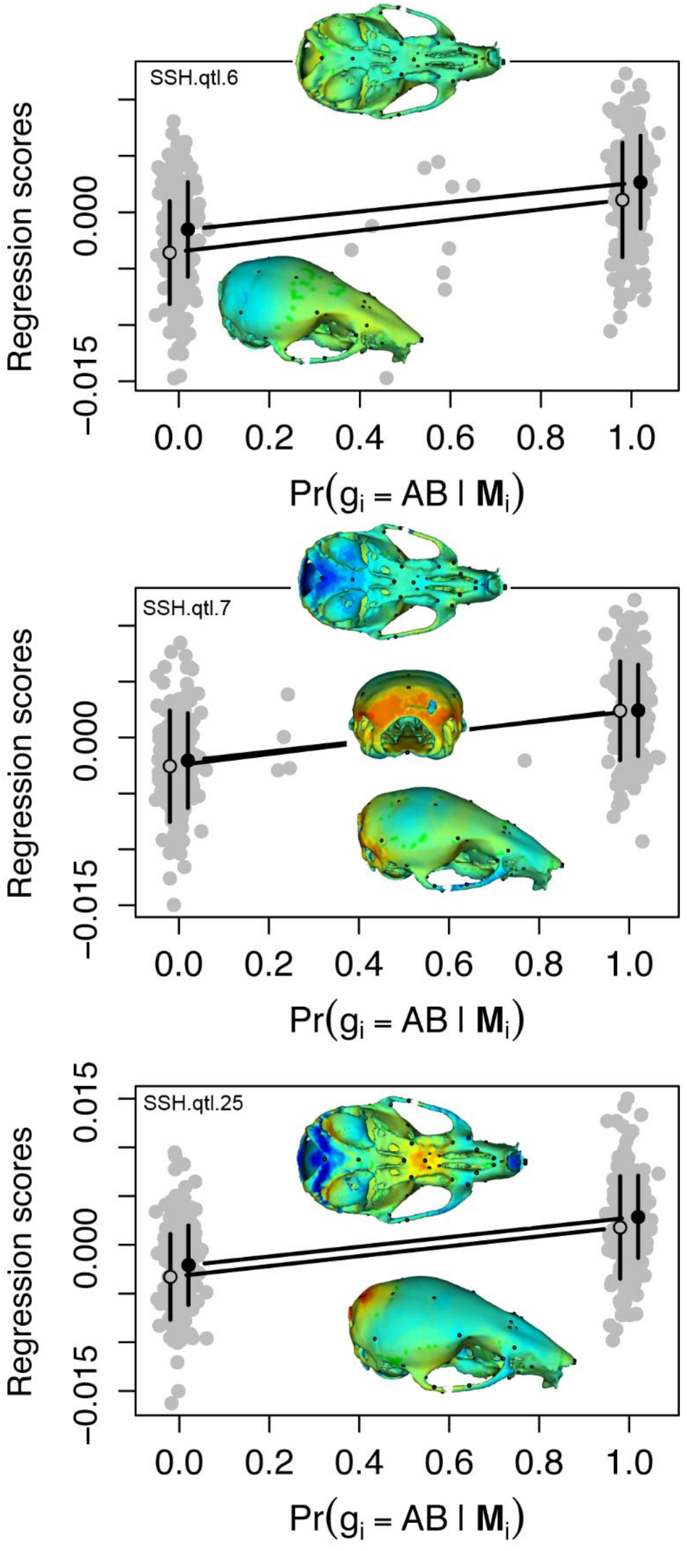

Supplemental Figure 2. Effect sizes of QTL. Effect sizes are expressed either as the amount of total Procrustes variance explained (*x*-axis) or for the *y*-axis as the proportion of variance explained of the shape variable the most associated with the QTL vector (projection scores; Drake and Klingenberg 2008). Each QTL is described by its chromosome and its position in centimorgans on this chromosome.

The amount of variation that each QTLs in the multiple QTL model accounts for, given other terms in the model (QTLs and covariates), was estimated as a percentage of the total Procrustes variance (Goodall 1991), a common measure used for association in geometric morphometrics (Monteiro, 1999). These effect sizes were also reported as a percentage of the projection score variance. Both measures of effect sizes are roughly correlated ($r_{spearman}$ = 0.54, p = 0.002), and have mainly the same right skewed distribution but they fundamentally differ in what they are looking at. The projection scores are the shape variable associated with the shape changes defined by the QTL and include both the effect and the residuals in that direction (Drake and Klingenberg 2008). Whereas the percentage of Procrustes variance is looking at the contribution of the QTL to the overall variation observed in skull shape, the percentage of the projection scores is looking at the contribution of the QTL to a specific aspect of shape variation. Thus, QTLs have individually a small contribution to the overall shape (< 1%) variation but they may account for 5% to 31.5% of the variation in those specific shape aspects, which are directions of the shape space that account for 3% to 6% of the overall shape variation (Supplementary Table 1).

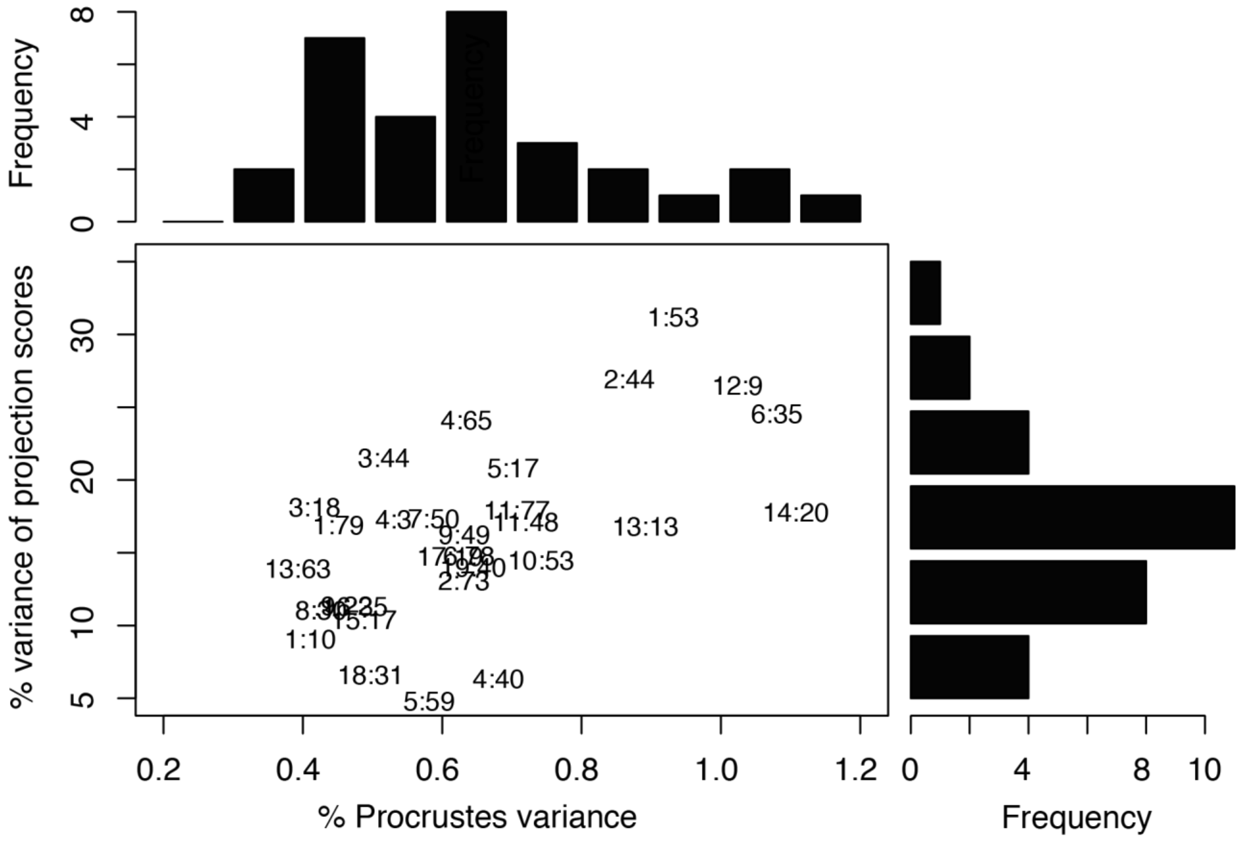

Supplementary Figure 3. Distribution of angles (°) between the QTL effects and the eigenvectors of the phenotypic matrix. a) Distribution of all pairs. The black curve describes the distribution of the angle between two random vectors of same dimension than the QTL effect. b) Correlation between the effect sizes of QTL measured as the magnitude of the QTL vector and the minimal angle between the QTL effect and the eigenvectors of the phenotypic matrix. QTL effects are no more similar to most PCs than two random vectors of the same dimension but the minimal angle between each QTL and PCs is. This minimal angle is nonetheless fairly high, between 50° and 70°, and correlates negatively with the magnitude of the QTL effect (r = -0.42, p = 0.02). Whereas the main axes of phenotypic variation did not coincide one to one with the QTL shape changes, they are related in some ways, and this agreement is higher when the magnitude is higher.

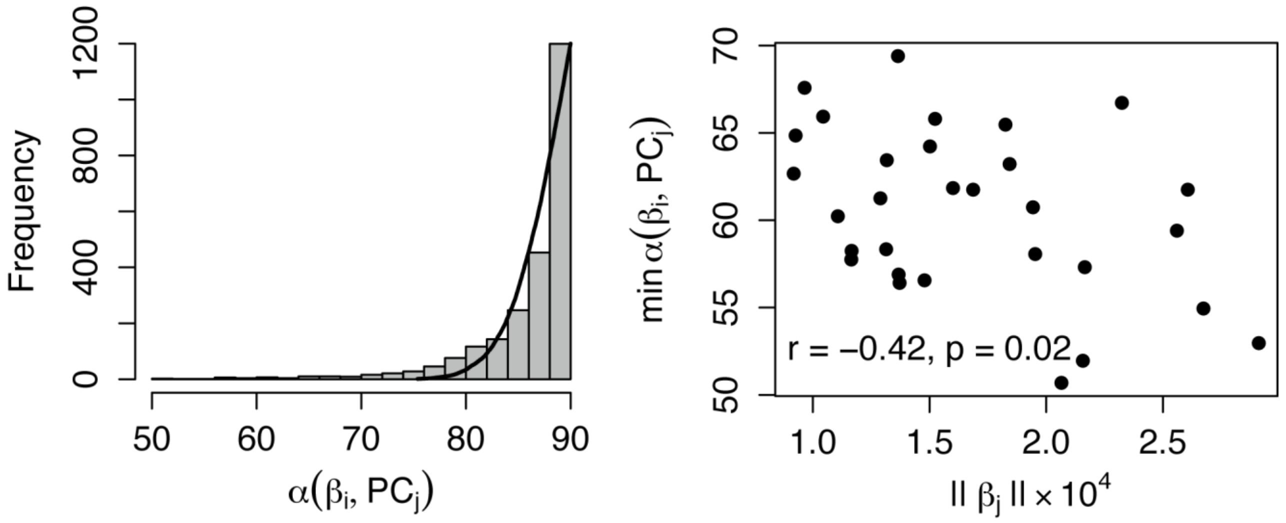

Supplementary Figure 4. Contribution of each anatomical region to the variation in covariates and SSH.qtl (top raw data, bottom normalized by the number of landmarks in each region). CS: Skull Centroid Size, D: Direction-of-cross, S: Sex, q1-q30: SSH.qtl.1-30

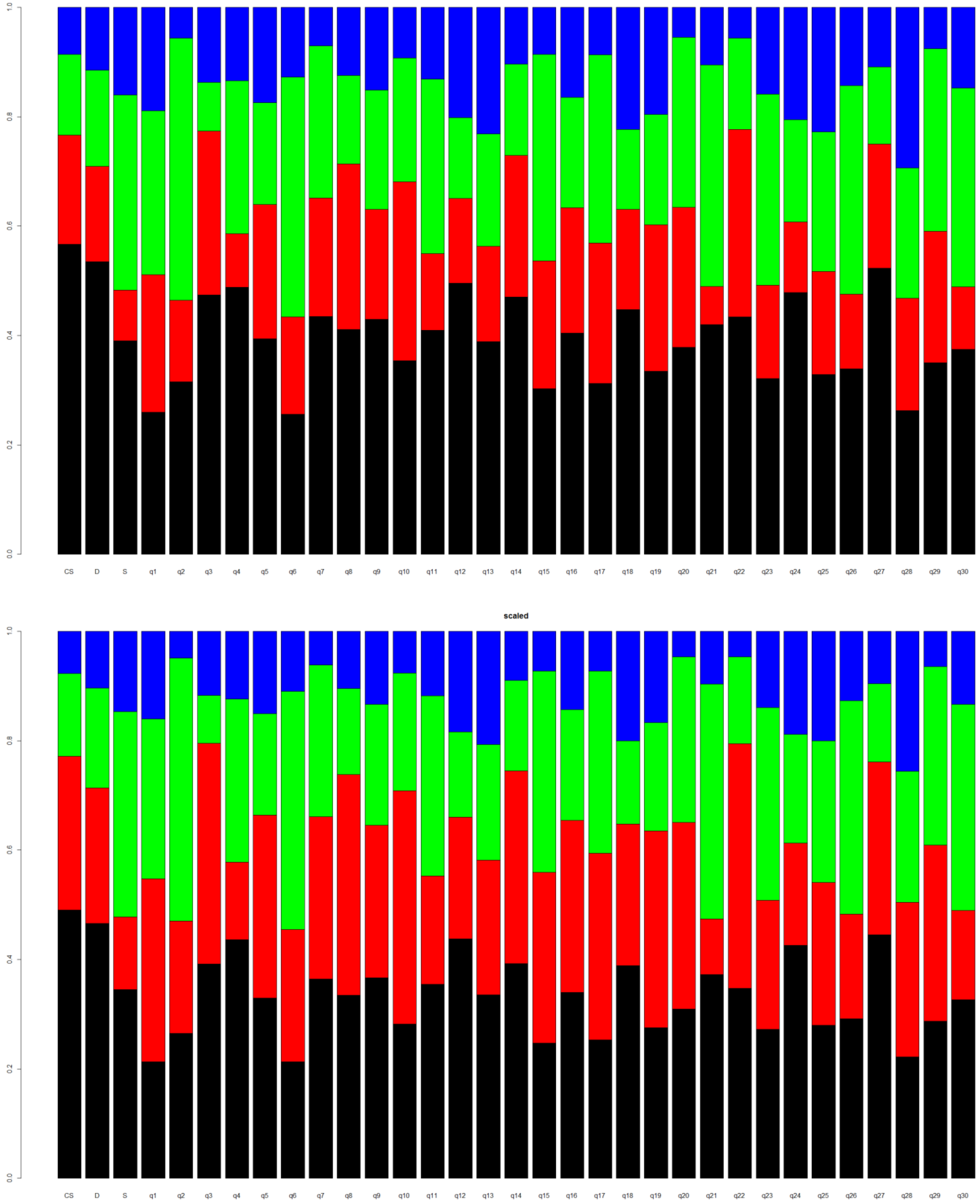

SUPPLEMENTAL TABLES

Supplemental Table. 1. Effect sizes of covariates and additive QTL of craniofacial shape.
1. The percentage of Procrustes variance explained by the effect given other QTLs and covariates.

2. The percentage of variance explained of the projection scores. The projection scores correspond to the shape variable the most correlated to the effect (Drake and Klingenberg 2008). In parenthesis is the percentage of Procrustes variance account by the projection scores. This is the actual amount of variance along the direction defined by the effect.

	Marker	$\|\|\beta\|\| \times 10^{-3}$	% shape variance[1]	% var. proj. scores[2]
**log(CS)**		255.25	4.19	59.74 (9.89)
**Dir. of cross**		7.57	2.27	48.13 (6.75)
**Sex**		4.33	0.82	17.37 (3.82)
**SSH.qtl1**	mCV24784983	3.04	0.40	9.37 (5.16)
**SSH.qtl2**	rs3678634	5.40	0.92	31.51 (5.40)
**SSH.qtl3**	rs13466711	3.69	0.44	17.18 (3.29)
**SSH.qtl4**	rs13480734	4.11	0.74	14.76 (5.16)
**SSH.qtl5**	rs13481127	4.65	0.71	17.41 (5.33)
**SSH.qtl6**	rs3672597	4.42	0.70	18.22 (4.92)
**SSH.qtl7**	rs3717860	4.82	1.04	26.78 (4.03)
**SSH.qtl8**	rs3710348	4.54	0.89	17.11 (5.81)
**SSH.qtl9**	gnf13.115.241	3.11	0.39	14.20 (3.29)
**SSH.qtl10**	rs6396829	5.17	1.10	18.06 (5.76)
**SSH.qtl11**	CEL-15_43206205	3.33	0.48	10.69 (4.40)
**SSH.qtl12**	rs4191367	3.23	0.46	11.63 (4.28)
**SSH.qtl13**	rs6298471	3.70	0.61	15.04 (4.50)
**SSH.qtl14**	rs6328845	3.41	0.49	6.93 (5.27)
**SSH.qtl15**	rs3023496	3.90	0.64	14.31 (4.08)
**SSH.qtl16**	rs13476580	5.06	0.86	27.21 (4.87)
**SSH.qtl17**	rs6209325	4.29	0.62	13.39 (4.09)
**SSH.qtl18**	rs6246699	3.41	0.41	18.44 (3.22)
**SSH.qtl19**	rs4138887	3.88	0.51	21.80 (3.99)
**SSH.qtl20**	rs3660863	3.59	0.51	17.60 (3.15)
**SSH.qtl21**	rs3711477	4.65	0.67	6.65 (6.19)
**SSH.qtl22**	rs3663950	4.41	0.62	24.39 (3.22)
**SSH.qtl23**	rs13459085	4.00	0.69	21.13 (4.09)
**SSH.qtl24**	CEL-5_117374791	3.70	0.57	5.11 (5.95)
**SSH.qtl25**	rs6181382	5.11	1.07	24.85 (4.96)
**SSH.qtl26**	rs6265387	3.84	0.63	15.10 (4.02)
**SSH.qtl27**	rs13479395	3.63	0.58	17.66 (3.80)
**SSH.qtl28**	rs13479776	3.03	0.41	11.36 (4.21)
**SSH.qtl29**	rs3714664	3.63	0.45	11.66 (4.07)
**SSH.qtl30**	rs13480351	4.27	0.62	16.53 (3.96)

Supplemental Table 2. List of craniofacial genes used as training set

ALX1
ALX3
ALX4
ANKH
ARHGAP31
ARID1A
ARID1B
ASXL1
CDON
CHD7
COL11A1
COL11A2
COL2A1
COL9A1
COL9A2
COLEC11
DHCR7
DHODH
DLX5
DLX6
DOCK6
EFNA4
EFTUD2
EOGT
ERF
EVC2
EYA1
EYA1
FCGR1A
FGFR1
FGFR2
FGFR3
FNDC3B
FOXE1
FOXL2
FRAS1
FRAS1
FREM1
FREM1
FREM2
FREM2
GDF6
GDF6
GLI2
GLI3
GNAI3
GNAS
GRHL3
GRIP1
GRIP1
HMX1
HOXA2
IFT122
IFT43
IGF1R
IRF6
KCNJ2
KMT2B
KMT2D
MASP1
MSX1
MSX2
MYH9
NME1
NME2
OTX2
PAX9
PIBF1
PLAG1
PLCB4
POLR1C
POLR1D
POR
PTCH1
PTPRF
PTPRS
PVRL1
RAB23
RBPJ
RECQL4
RUNX2
SALL1
SALL4
SF3B4
SH3BP2
SHH
SIX1
SIX3
SIX5
SKI
SLC26A2
SMARCA4
SMARCB1
SMARCE1
SOX9
TCF12
TCOF1
TFAP2A
TGFB3
TGFBR1
TGFBR2
TGIF1
TP63
TSHZ1
TWIST1
WDR35
WNT9B
